# Association of secondhand smoke exposure with cardiometabolic health in never-smoking adult cancer survivors: a population-based cross-sectional study

**DOI:** 10.1186/s12889-022-12962-y

**Published:** 2022-03-17

**Authors:** Kyuwoong Kim, Yoonjung Chang

**Affiliations:** 1 Division of Cancer Control and Policy, National Cancer Control Institute, National Cancer Center, Goyang, Gyeonggi-do Republic of Korea; 2 Department of Cancer Control and Population Health, Graduate School of Cancer Science and Policy, National Cancer Center, Goyang, Republic of Korea

**Keywords:** Cancer survivors, Secondhand smoking, Cardiometabolic risk factors

## Abstract

**Background:**

Little is known about the association of secondhand smoke (SHS) exposure with cardiometabolic health in adult cancer survivors, especially those who have never smoked. This study aimed to investigate the association of SHS exposure and cardiometabolic health in never-smoking adult cancer survivors.

**Methods:**

Cross-sectional data of 830 adult cancer survivors aged more than 19 years who were never-smokers were identified from the Korea National Health and Nutrition Survey (KNHANES) 2013–2018, a nationally representative sample of the noninstitutionalized Korean population. SHS exposure was defined from self-reported survey and cardiometabolic outcomes (hypertension, general and abdominal obesity, hyperlipidemia, hypertriglyceridemia, reduced high-density lipoprotein, and impaired fasting glucose) were determined according to relevant criteria and data from the KNHANES. We used multiple logistic regression to compute odds ratio (OR) and 95% confidence intervals (95% CI) comparing those with and without SHS exposure for each outcome adjusted for potential confounders.

**Results:**

Compared with the never-smoking adult cancer survivors without SHS exposure, those with SHS exposure had significantly higher odds for hypertriglyceridemia (OR = 1.63; 95% CI: 1.07–2.48). However, the other outcomes showed nonsignificant associations with SHS exposure (hypertension [OR = 1.33; 95% CI: 0.90–1.96]. general obesity [OR = 1.47; 95% CI: 1.47: 0.97–2.22], abdominal obesity [OR = 1.20; 95% CI: 0.82–1.75], hyperlipidemia [OR = 1.03; 95% CI: 0.68–1.55], reduced HDL-cholesterol [OR = 1.01; 95% CI: 0.70–1.45], and impaired fasting glucose [OR = 1.07; 95% CI: 0.72–1.58].

**Conclusion:**

This cross-sectional study suggests the association of SHS exposure with hypertriglyceridemia and provides evidence for marginal associations with other cardiometabolic risk factors in never-smoking adult cancer survivors. More studies are needed to develop evidence-based public health policies to minimize SHS exposure in adult cancer survivors.

**Supplementary Information:**

The online version contains supplementary material available at 10.1186/s12889-022-12962-y.

## Background

**Fig. 1:**
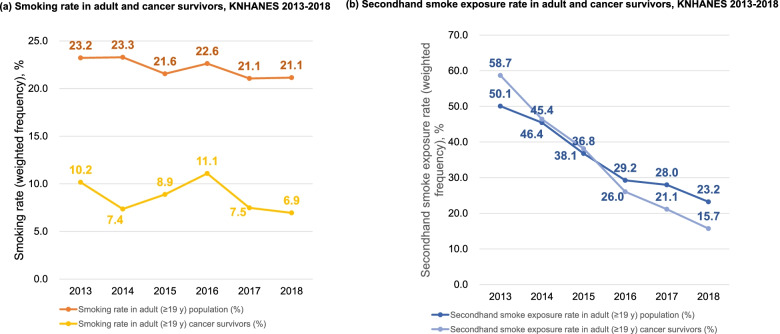
Trends in smoking rate and secondhand smoke exposure in the general adult population and adult cancer survivors in the KNHANES 2013–2018. Adult is defined as those older than 19 years of age. Acronym: KNHANES, Korea National Health and Nutrition Examination Survey

Secondhand smoke (SHS) exposure is a consistent and serious public health problem worldwide, especially for the vulnerable population such as infants, children, and pregnant women as well as cancer survivors [1–7]. In the United States, SHS exposure among non-smokers and community-dwelling adult cancer survivors are reported to be on the decline [8, 9] and similar trends are observed in the Republic of Korea based on the U.S National Health and Nutrition Examination Survey (NHANES) and Korea National Health and Nutrition Examination Survey (KNHANES) (Fig. 1), respectively. However, SHS exposure is still prevalent up to approximately 15% among the adult cancer survivors in the U.S and Korean population. In addition, adult cancer survivors may need additional clinical attention for the management of cardiometabolic health to prevent adverse health outcomes such as cancer recurrence, secondary primary cancer (SPC), cardiovascular disease (CVD), and all-cause mortality (ACM) as well as biomarkers of CVD [10–16].

Some studies indicate that SHS exposure is associated with worsening of cardiometabolic health [17–22]. However, there is not enough evidence on the association of SHS exposure and cardiometabolic health in adult cancer survivors whose cardiometabolic health is of clinical importance for prevention of adverse health outcomes. In addition, the National Comprehensive Cancer Network (NCCN) Guidelines for Smoking Cessation suggests that patients with cancer should stop the use of all kinds of tobacco products for substantial health benefits [23]. Nonetheless, SHS exposure is not a risk-free factor for cancer survivors who are lifetime never-smokers. While the extent to which SHS exposure is associated with cardiometabolic health in never-smoking cancer survivors is unclear, reducing adverse health effects from SHS exposure still remains as an important public health challenge.

From 2013 to 2018, information on SHS exposure and cardiometabolic health in never-smoking adult cancer survivors were collected as a part of KNHANES among a nationally representative sample of non-institutionalized Korean population. We hypothesized that the SHS exposure is associated with higher odds of worsened cardiometabolic health in never-smoking adult cancer survivors.

## Methods

### Study population

**Fig. 2:**
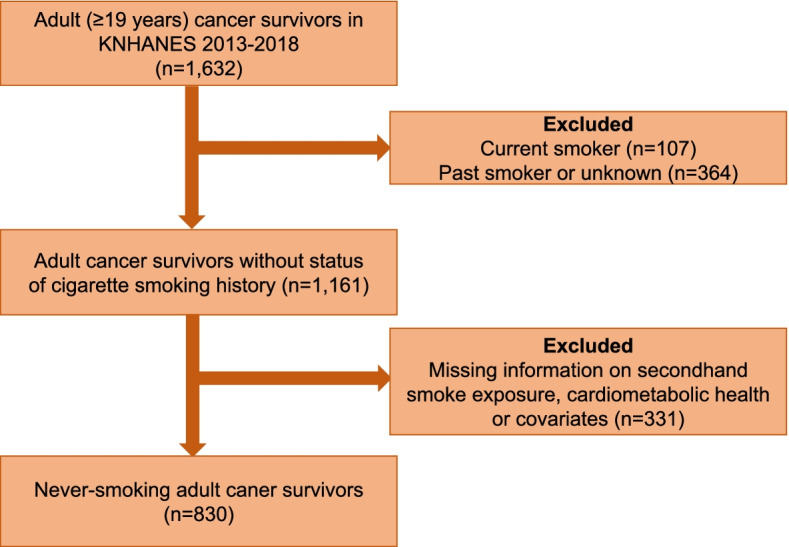
Study population selection from KNHANES (Korea National Health and Nutrition Examination Survey) 2013–2018 to investigate the association of secondhand smoke exposure and cardiometabolic health in never-smoking adult cancer survivors

KNHANES is a nationally representative cross-sectional study conducted by the Korea Disease Control Agency (KDCA) and the database has been annually collected from the non-institutionalized men and women aged more than1 year since 1998. Details of the KNHANES have been previously described and published elsewhere [24]. Briefly, a complex, multistage probability sampling design was used in the KNHANES to include a representative sample of the Korean population. The participants in this study were never-smoking adult (≥ 19 years) cancer survivors enrolled from the sixth and seventh KNHANES (2013–2018) with history of any type of cancer based on the self-reported questionnaire on cigarette smoking status and cancer diagnosis records, respectively. Of the 1,161 never-smoking adult cancer survivors, we excluded 331 participants with missing information on SHS exposure, cardiometabolic health or covariates to investigate the association of SHS exposure with cardiometabolic health in never-smoking adult cancer survivors. The final study population included 830 never-smoking adult cancer survivors (Fig. 2). The Institutional Review Board (IRB) of KCDA, which is in accordance with the guidelines of the Declaration of Helsinki approved the protocols of the research and data release for the KNHANES (2013-12EXP-03-5C). Prior to participating in the KNHANES, all subjects provided informed consent. The KCDA grants researchers access to the KNHANES database for research purpose upon approval (www.kdca.go.kr).

### Secondhand smoke (SHS) exposure

Information on SHS for never-smoking adult cancer survivors was derived from the following self-report questionnaires in the KNHANES: “During the past week, were you ever exposed to SHS at work?”, “During the past week, were you ever exposed to SHS at home?”, and “During the past week, were you ever exposed to SHS at public places (e.g. public institution buildings, schools, libraries, public transportation, concert halls, tourist accommodations, restaurants, and etc.) except for the designated smoking areas?” Based on the responses to the self-report questionnaires, SHS exposure was defined as any SHS exposure at work, home or in public place. Assessment of SHS status from the self-report questionnaires in the KNHANES has been described in previous studies [25, 26].

### Components of cardiometabolic health

Based on the blood pressure and anthropometric measurements, blood samples (after at least 8 h of fasting) and self-report measures on medication in never-smoking adult cancer survivors, cardiometabolic health were categorized as the following: hypertension (2017 American Heart Association, AHA/American College of Cardiology, ACC criteria), general and abdominal obesity (Korean Society for the Study of Obesity criteria modified from the World Health Organization definition for the Asian population), hyperlipidemia, hypertriglyceridemia, reduced high density lipoprotein cholesterol (HDL-C), and impaired fasting glucose (American Diabetes Association, ADA/World Health Organization, WHO criteria) as suggested in previous studies [27–30]. Hypertension (2017 AHA/ACC) was defined as systolic blood pressure (SBP) ≥ 130 mmHg and diastolic blood pressure (DBP) ≥ 80 mmHg or taking antihypertensive drugs. General and abdominal obesity was defined as body mass index (BMI) ≥ 25.0 kg/m^2^ and waist circumference (WC) ≥ 90 cm for men and WC ≥ 85 cm for women, respectively. Hyperlipidemia, hypertriglyceridemia, and reduced HDL-C were defined as total cholesterol ≥ 240 mg/dL or taking cholesterol lowering drugs, triglyceride ≥ 200 mg/dL or taking cholesterol lowering drugs, and HDL-C ≤ 40 mg/dL for men and ≤ 50 mg/dL for women, respectively. Impaired fasting glucose was defined as fasting serum glucose of 100–125 mg/dL.

### Other variables

Self-report questionnaires and 24-h dietary recall in the KNHANES were used to obtain sociodemographic factors (age, sex, education level, household income, insurance type, marital status, residential area, occupation type), health behavior (regular aerobic exercise, muscle strengthening exercise, and alcohol consumption), dietary intake (total energy intake), cancer sites, and family history of cardiovascular disease (coronary heart disease and total stroke). Education level was defined as the highest education completed and was categorized as elementary school, middle school, high school, and ≥ college/university. Household income was calculated by dividing the sum of the income contributed from the total household members by the square root of the number of household members and categorized into quartiles. Insurance type was categorized as self-employed insured, employee insured, and medical aid according to the nationwide health insurance system (i.e. National Health Insurance Service) implemented in the Republic of Korea. Marital status was categorized as married and single (no history of marriage), widowed, divorced, or separated. Residential area was categorized into urban/metropolitan and rural based on the administrative units of the participants according to a previous study. Occupation type was categorized as manager, professional, manual labor, or unemployed based on the classification of job roles from a previous study. Physical activity was classified as regular aerobic exercise (engaging in at least 1.25 to 2.5 h of moderate to vigorous aerobic exercise per week) and muscle strengthening exercise (categorized as none, 1–2 times, 3–4 times, and ≥ 5 times per week) in accordance to the *Physical Activity Guidelines for Americans, 2*^*nd*^* edition *[31]. Alcohol consumption was categorized into none (non-drinkers) and habitual alcohol consumption (consuming at least one standard drink, which is equal to 10.0 g of alcohol). Cancer sites were classified as gastric cancer, colorectal cancer, breast cancer, cervical cancer, thyroid cancer, and other types of cancer (i.e. other than the above-mentioned cancer types) according to the self-report questionnaire [32]. Family history of cardiovascular disease was defined as family history of coronary heart disease or total stroke.

### Statistical analysis

Survey regression analysis and Rao-Scott F-adjusted chi-square tests were used to compare the characteristics (sociodemographic factors, health behavior, dietary intake, cancer sites, and family history of cardiovascular disease as continuous and categorical variables) for never-smoking adult cancer survivors according to SHS exposure status. These characteristics of the participants were calculated using mean (standard error) for continuous variables and number (weighted percentage) for categorical variables using sampling weights in the KNHANES. For each dichotomous outcome of hypertension (2017 AHA/ACC), general obesity, abdominal obesity, hyperlipidemia, hypertriglyceridemia, reduced HDL-C, and impaired fasting glucose (ADA/WHO), we used multivariable logistic regression adjusted for sociodemographic factors (age, sex, education level, household income, marital status, residential area, and occupation type) for Model 1 to estimate the odds ratio (OR) and 95% confidence intervals (95% CI) for association of SHS exposure with cardiometabolic health. In addition, Model 2 was constructed from adding health behaviors (aerobic exercise, muscle strengthening exercise, alcohol consumption, and total energy intake) into the variables included in Model 1 and Model 3 was additionally adjusted for family history of cardiovascular disease including variables from the Model 2. All of the regression models were accounted for sampling weights in the KNHANES for the participants. In addition, we carried out the F-adjusted mean residual goodness-of-fit test and tested multicollinearity between the independent variables for each model (Supplemental Table 1).

For subgroup analyses, we stratified the participants into age (< 65 years and ≥ 65 years), sex (male and female), education level (university/college and < high school), household income (upper half and lower half), and occupation status (manager, professional, manual labor, and unemployed) and conducted multivariable logistic regression to investigate the association of SHS exposure and components of cardiometabolic health in each subgroup included in Model 3 using the sampling weights. In addition, we performed log likelihood test for interaction effects for variables used in the subgroup analyses. Data collection and statistical analyses were performed with SAS software version 9.4 (SAS Institute., NC, USA). *P*-values were two-sided and *p*-values less than 0.05 was considered statistically significant.

## Results

### Characteristics of participants

In this study, approximately one third of the community-dwelling adult cancer survivors were classified as those with SHS exposure regardless of the declining trend of SHS exposure in both general population and cancer survivors in the KNHANES, 2013–2018. Adult cancer survivors with SHS exposure was younger and slightly higher proportion of them male, without habitual alcohol consumption, residing in urban or metropolitan area, and were working in manual labor compared to those without SHS exposure (Table 1). In addition, marital status, energy intake, cancer sites, family history of cardiovascular diseases and other characteristics did not differ by SHS exposure in adult cancer survivors.

**Table 1:** Characteristics of adult cancer survivors in the Korea National Health and Nutrition Examination Survey, 2013–2018 by secondhand smoke exposure status

	**No Secondhand Smoke Exposure in Never-Smoking Adult Cancer Survivors** **(*N* = *591*)**	**Secondhand** **Smoke Exposure in Never-Smoking Adult Cancer Survivors** **(*N* = *239*)**	***P*-value**^**a**^
Age, mean (SE), years	59.4 (0.58)	54.6 (1.13)	< 0.001
Sex			0.015
Male	52 (8.8)	27 (11.3)	
Female	539 (91.2)	212 (88.7)	
Education level			0.462
Elementary school	228 (38.7)	73 (30.5)	
Middle School	83 (14.1)	39 (16.3)	
High School	150 (25.5)	71 (29.7)	
≥ College/University	128 (21.7)	56 (23.4)	
Household income			0.045
1Q	169 (28.6)	58 (24.3)	
2Q	137 (23.2)	71 (29.7)	
3Q	137 (23.2)	65 (27.2)	
4Q	148 (76.7)	45 (23.3)	
Insurance type			0.003
Self-employed insured	165 (27.9)	93 (38.9)	
Employee insured	397 (67.2)	139 (58.2)	
Medical aid	28 (4.7)	7 (2.9)	
Marital status			0.01
Married	582 (98.5)	231 (96.7)	
Single, widowed, divorced, separated	9 (1.5)	8 (3.4)	
Residential area			0.599
Urban/Metropolitan	419 (70.9)	175 (73.2)	
Rural	172 (29.1)	64 (26.8)	
Occupation type			0.034
Manager, professionals	64 (10.8)	35 (14.6)	
Manual labor	156 (26.4)	82 (34.3)	
Unemployed	371 (62.8)	122 (51.1)	
Regular aerobic exercise^b^	218 (36.9)	89 (37.2)	0.438
Muscle strengthening exercise			0.023
None	489 (82.7)	179 (74.9)	
1–2 times per week	32 (5.4)	29 (12.1)	
3–4 times per week	36 (6.1)	10 (4.2)	
≥ 5 times per week	34 (5.8)	21 (8.8)	
Alcohol consumption			< 0.001
None	448 (75.8)	146 (61.1)	
Habitual consumption^c^	143 (24.2)	93 (38.9)	
Total energy intake (kcal/day), mean (SE)	1021.5 (32.9)	1005.0 (51.2)	0.793
Cancer sites			
Gastric cancer	79 (13.4)	25 (10.5)	0.698
Colorectal cancer	48 (8.1)	16 (6.7)	0.025
Breast cancer	113 (19.1)	48 (20.1)	0.670
Cervical cancer	93 (15.7)	42 (17.6)	0.309
Thyroid cancer	169 (28.6)	65 (27.2)	0.820
Other types	100 (16.9)	43 (18.0)	0.448
Family history of cardiovascular disease^d^	113 (19.1)	48 (20.1)	0.284

Values above are presented as n (%) using unless otherwise specified

*Abbreviations: SE* Standard Error, *Q* Quartile

^a^Computed from survey regression for continuous variables and Rao-Scott F-adjusted chi-square test for categorical variables

^b^Engaging in at least 1.25 to 2.5 h of moderate to vigorous aerobic exercise per week

^c^Consuming at least one standard drink (approximately 10.0 g of alcohol) in a month

^d^Family history of coronary heart disease or stroke

### SHS and cardiometabolic health in never-smoking adult cancer survivors

In the fully adjusted model including sociodemographic factors (age, sex, education level, household income, marital status, residential area, and occupation type), health behavior (aerobic exercise, muscle strengthening exercise, alcohol consumption, and total energy intake), and family history of cardiovascular disease, the adult cancer survivors with SHS exposure generally had higher odds for hypertension according to 2017 AHA/ACC criteria (OR = 1.33; 95% CI: 0.90–1.96), general obesity (OR = 1.47; 95% CI: 0.97–2.22), abdominal obesity (OR = 1.20; 95% CI: 0.82–1.75), hyperlipidemia (OR = 1.03; 95% CI: 10.68–1.55), hypertriglyceridemia (OR = 1.63; 95% CI: 1.07–2.48), reduced HDL-C (OR = 1.01; 95% CI: 0.70–1.45), and impaired fasting glucose according to ADA/WHO criteria (OR = 1.07; 95% CI: 0.72–1.58) compared to those without SHS exposure (Table 2).

**Table 2:** Association of secondhand smoke exposure with cardiometabolic health among never-smoking adult cancer survivors in the Korea National Health and Nutrition Examination Survey, 2013–2018

	**No Secondhand Smoke Exposure in Never-Smoking Adult Cancer Survivors** **(*N* = *591*)**	**Secondhand** **Smoke Exposure in Never-Smoking Adult Cancer Survivors** **(*N* = *239*)**
**Hypertension (2017 AHA/ACC**^**a**^**)**		
Model 1	1.00 (Ref)	1.29 (0.88–1.89)
Model 2	1.00 (Ref)	1.31 (0.89–1.93)
Model 3	1.00 (Ref)	1.33 (0.90–1.96)
**General obesity**^**b**^		
Model 1	1.00 (Ref)	1.47 (0.98–2.22)
Model 2	1.00 (Ref)	1.47 (0.97–2.23)
Model 3	1.00 (Ref)	1.47 (0.97–2.22)
**Abdominal obesity**^**b**^		
Model 1	1.00 (Ref)	1.20 (0.82–1.75)
Model 2	1.00 (Ref)	1.19 (0.81–1.74)
Model 3	1.00 (Ref)	1.20 (0.82–1.75)
**Hyperlipidemia**^**c**^		
Model 1	1.00 (Ref)	0.99 (0.67–1.47)
Model 2	1.00 (Ref)	1.02 (0.67–1.54)
Model 3	1.00 (Ref)	1.03 (0.68–1.55)
**Hypertriglyceridemia**^**c**^		
Model 1	1.00 (Ref)	1.63 (1.08–2.46)^*^
Model 2	1.00 (Ref)	1.62 (1.06–2.47)^*^
Model 3	1.00 (Ref)	1.63 (1.07–2.48)^*^
**Reduced HDL-C**^**c**^		
Model 1	1.00 (Ref)	1.04 (0.72–1.49)
Model 2	1.00 (Ref)	0.99 (0.69–1.43)
Model 3	1.00 (Ref)	1.01 (0.70–1.45)
**Impaired fasting glucose (ADA/WHO**^**d**^**)**		
Model 1	1.00 (Ref)	1.07 (0.73–1.58)
Model 2	1.00 (Ref)	1.06 (0.71–1.57)
Model 3	1.00 (Ref)	1.07 (0.72–1.58)

Values above are presented as adodds ratios and 95% confidence intervals computed from multiple logistic regression accounting for the sampling weights. Model 1 was adjusted for sociodemographic factors (age, sex, education level, household income, marital status, residential area, occupation type), Model 2 was adjusted for variables included in Model 1 and health behavior (aerobic exercise, muscle strengthening exercise, alcohol consumption, total energy intake), and Model 3 was adjusted for family history of cardiovascular disease (coronary heart disease or stroke) in addition to the variables included in Model 1 and 2

*Acronyms: AHA* American Heart Association, *ACC* American College of Cardiology, *BMI* Body Mass Index, *WC* Waist Circumference, *HDL-C* High-Density Lipoprotein Cholesterol, *ADA* American Diabetes Association, *WHO* World Health Organization, *SBP* Systolic Blood Pressure, *DBP* Diastolic Blood Pressure, *FSG* Fasting Serum Glucose

^a^Defined as SBP ≥ 130 mmHg or DBP ≥ 80 mmHg or taking antihypertensive drugs according to the 2017 AHA/ACC high blood pressure guidelines

^b^General obesity defined as BMI ≥ 25.0 kg/m^2^ and abdominal obesity defined as WC ≥ 90 cm for men and WC ≥ 85 cm for women

^c^Hyperlipidemia defined as total cholesterol ≥ 240 mg/dL or taking cholesterol lowering drugs; hypertriglyceridemia defined as triglyceride ≥ 200 mg/dL or taking cholesterol lowering drugs: reduced HDL-C defined as HDL-C ≤ 40 mg/dL for men and HDL-C ≤ 50 mg/dL for women

^d^Defined as FSG of 110–125 mg/dL according to the ADA and WHO

^*^p < 0.05

### Subgroup analysis

The overall results in the subgroup analyses across the sociodemographic variables (age, sex, education level, household income, and occupation type) showed similar results to the main analysis for each component of cardiometabolic health (Table 3). Statistically significant association of SHS exposure with obesity in never-smoking adult cancer survivors were found for general obesity among those in lower half of household income (OR = 1.77; 95% CI: 1.06–2.96) and those working manual labor (OR = 2.62; 95% CI: 1.38–4.97) compared to never-smoking adult cancer survivors without SHS exposure. Compared to the never-smoking adult cancer survivors without SHS exposure, those with SHS exposure who were older adults (≥ 65 years), male, and upper half of household income had higher odds for hypertriglyceridemia. Similar trend was observed in older adults (≥ 65 years) and lower half of household income for reduced HDL-C in never-smoking adult cancer survivors compared to those without SHS exposure. Interaction testing in the multivariable model showed difference in association of SHS exposure on cardiometabolic health within subgroups of age, education level, and household income (interaction *p-values* < 0.05 for all comparisons) (Supplemental Table 2).

**Table 3:** Stratified analysis for cardiometabolic health among never-smoking adult cancer survivors with secondhand smoke exposure compared to those without in the Korea National Health and Nutrition Examination Survey, 2013–2018

	**Hypertension (2017 AHA/ACC**^**a**^**)**	**General obesity**^**b**^	**Abdominal obesity**^**b**^	**Hyperlipidemia**^**c**^	**Hypertriglyceridemia**^**c**^	**Reduced** **HDL-C**^**c**^	**Impaired fasting glucose (ADA/WHO**^**d**^**)**
Age							
< 65 y	1.60 (1.00–2.56)^*^	1.45 (0.85–2.45)	1.22 (0.76–1.94)	0.88 (0.53–1.46)	1.41 (0.79–2.51)	0.81 (0.52–1.26)	1.54 (0.92–2.56)
≥ 65 y	0.65 (0.32–1.30)	1.42 (0.74–2.75)	1.04 (0.55–1.97)	1.24 (0.65–2.37)	2.54 (1.26–5.12)^*^	2.17 (1.20–3.93)^*^	0.65 (0.35–1.20)
Sex							
Male	2.77 (0.99–7.75)	2.79 (0.79–9.88)	2.39 (0.48–12.08)	1.13 (0.17–7.55)	5.36 (1.38–20.74)^*^	2.85 (0.86–9.46)	0.86 (0.28–2.69)
Female	1.22 (0.82–1.83)	1.27 (0.82–1.96)	1.07 (0.72–1.60)	0.96 (0.62–1.47)	1.37 (0.89–2.11)	0.98 (0.68–1.43)	1.07 (0.70–1.63)
Education level							
University/College	1.53 (0.88–2.66)	1.43 (0.79–2.58)	1.53 (0.88–2.65)	0.89 (0.50–1.58)	1.82 (0.97–3.40)	1.01 (0.61–1.64)	1.97 (1.06–3.67)
≤ High school	1.05 (0.60–1.83)	1.57 (0.87–2.83)	0.93 (0.53–1.64)	1.37 (0.73–2.57)	1.85 (1.02–3.33)^*^	1.05 (0.62–1.79)	0.67 (0.39–1.14)
Household income							
Upper half	1.51 (0.83–2.76)	1.24 (0.62–2.45)	1.08 (0.62–1.87)	1.71 (0.93–3.16)	1.94 (1.07–3.50)^*^	0.61 (0.35–1.06)	1.29 (0.70–2.38)
Lower half	1.32 (0.80–2.20)	1.77 (1.06–2.96)^*^	1.43 (0.84–2.41)	0.71 (0.41–1.22)	1.41 (0.79–2.51)	1.92 (1.16–3.18)^*^	0.90 (0.52–1.56)
Occupation type							
Manager, professionals	1.99 (0.71–5.57)	0.69 (0.20–2.44)	1.08 (0.35–3.38)	0.71 (0.18–2.75)	2.10 (0.64–6.87)	0.88 (0.26–2.98)	0.98 (0.27–3.64)
Manual labor	1.90 (0.97–3.72)	2.62 (1.38–4.97)^*^	1.46 (0.78–2.73)	1.11 (0.57–2.15)	1.68 (0.88–3.21)	1.20 (0.63–2.29)	1.69 (0.93–3.07)
Unemployed	0.94 (0.56–1.58)	1.23 (0.69–2.18)	1.11 (0.66–1.88)	1.00 (0.57–1.72)	1.54 (0.85–2.76)	1.04 (0.63–1.71)	0.89 (0.54–1.47)

Values above are presented as adjusted odds ratios and 95% confidence intervals computed from multiple logistic regression accounting for the sampling weights. Model 1 was adjusted for sociodemographic factors (age, sex, education level, household income, marital status, residential area, occupation type), health behavior (aerobic exercise, muscle strengthening exercise, alcohol consumption, total energy intake), and family history of cardiovascular disease (coronary heart disease or stroke). Interaction *p*-values for each outcome are noted in Supplemental Table 2

*Acronyms: AHA* American Heart Association, *ACC* American College of Cardiology, *BMI* Body Mass Index, *WC* Waist Circumference, *HDL-C* High-Density Lipoprotein Cholesterol, *ADA* American Diabetes Association, *WHO* World Health Organization, *SBP* Systolic Blood Pressure, *DBP* Diastolic Blood Pressure, *FSG* Fasting Serum Glucose

^a^Defined as SBP ≥ 130 mmHg or DBP ≥ 80 mmHg or taking antihypertensive drugs according to the 2017 AHA/ACC high blood pressure guidelines

^b^General obesity defined as BMI ≥ 25.0 kg/m^2^ and abdominal obesity defined as WC ≥ 90 cm for men and WC ≥ 85 cm for women

^c^Hyperlipidemia defined as total cholesterol ≥ 240 mg/dL or taking cholesterol lowering drugs; hypertriglyceridemia defined as triglyceride ≥ 200 mg/dL or taking cholesterol lowering drugs: reduced HDL-C defined as HDL-C ≤ 40 mg/dL for men and HDL-C ≤ 50 mg/dL for women

^d^Defined as FSG of 110–125 mg/dL according to the ADA and WHO

^*^p < 0.05

## Discussion

In this population-based cross-sectional study of never-smoking adult cancer survivors, SHS exposure was significantly associated with higher odds of hypertriglyceridemia. Findings of this study showed that SHS exposure was marginally associated with hypertension, general obesity, abdominal obesity, hyperlipidemia, reduced HDL-C, and impaired fasting glucose. These associations were only statistically significant in certain subgroups, particularly by age, education level, household income, and occupation type.

The overall decreasing trend in SHS exposure found in this study is comparable to other studies conducted in the United States [8, 9]. Among the adult general population and adult cancer survivors in the KNHANES 2013–2018, SHS exposure decreased from 58.7% and 50.1% in 2013 to 23.2% and 15.7%, respectively. Compared to this, SHS exposure in nonsmokers declined from 87.5% to 25.2% from 1988 to 2014 and SHS exposure in nonsmoking adult cancer survivors is reported to be 28.3% between 1999 and 2012 in the United States based on the NHANES data. Among the community-dwelling adult cancer survivors in the U.S, those in the certain ethnic group, low socioeconomic status, and history of smoking-related cancer were more likely to be exposed to SHS [9]. Nonetheless, SHS exposure is still prevalent in never-smoking adult cancer survivors in both KNHANES and NHANES. In addition, such trends do not necessarily represent that the adverse health outcomes associated with SHS is also on the decline for the never-smoking adult cancer survivors.

Our finding of the association between SHS exposure and cardiometabolic health in never-smoking adult cancer survivors was generally consistent with previous studies, which mostly investigated the association between SHS exposure and cardiometabolic health in the general population. In the Kangbuk Samsung Cohort study (KSCS), a longitudinal cohort study of Korean men and women over 18 years of age showed significant association of SHS exposure with hypertension in never-smokers who were exposed via passive smoking in both home and workplace [33]. Secondhand smoking was associated with higher odds of obesity and glucose abnormalities as compared to non-smokers in the NHANES 1999–2010 as well as worsening of lipid profile in a mouse model for human apolipoprotein (ApoB100) [20, 34]. While these studies were conducted in the general population such as KSCS and NHANES or in a laboratory setting, our study focused on never-smoking adult cancer survivors who are reported to have higher cardiovascular risk compared to those without history of cancer. In our analyses, significant association of SHS exposure and hypertension in never-smoking adult cancer survivors was observed, but limited statistical evidence was found for obesity, worsening of lipid profile, and glucose abnormalities. This may potentially due to variations in SHS exposure as a measure of intensity and duration of passive smoking history and consistent exposure in each study, which is difficult to accurately quantify based on self-reported survey data. Currently, there is no clear evidence on the extent to which SHS exposure contributes to worsening of cardiometabolic health in adult cancer survivors, especially those who are never-smokers.

Some evidence might partially explain the findings of our study. SHS contains same harmful chemicals such as carcinogens (e.g. benzene, vinyl chloride, and formaldehyde), toxic metals (e.g. arsenic and cadmium) and poisons (e.g. carbon monoxide) that active cigarette smokers inhale [35]. Besides active smoking, SHS exposure alone could negatively influence blood pressure, BMI, lipid profile, and glucose level for the adult cancer survivors who never smoked through the same physiological mechanisms as the previous studies have shown [1, 18, 20, 22, 26, 36]. In addition, findings from the ATTICA study showed that SHS exposure is associated with higher levels of inflammatory markers with previous evidence for cardiovascular risk such as white blood cell, C-reactive protein, homocysteine, fibrinogen, and oxidized low-density lipoprotein cholesterol in never-smoking men and women without history of cardiovascular disease [37]. Taken this evidence together, protection of never-smokers from SHS exposure, especially for those with history of cancer should be promoted despite the difference in magnitude of statistical evidence found in each study.

### Study strengths and limitations

The most notable strength of this study is the availability of SHS exposure data among community-dwelling adult cancer survivors who were never-smokers to examine the association of SHS exposure with cardiometabolic health. Limiting the study population to never-smoking cancer survivors minimizes the confounding effect from active smoking on cardiometabolic health. Also, our findings add evidence for an association of SHS exposure on cardiometabolic health in never-smoking adult cancer survivors, suggesting public health necessity for minimizing SHS exposure for this vulnerable population. However, SHS exposure was collected from a self-reported questionnaire in the KNHANES, which is subject to recall bias. In addition, information on intensity and duration of SHS exposure was not available for further assessment. Previous literature also indicated the difficulty of accurately measuring SHS exposure, including the assessment through relevant biomarker. Most of the studies that examined SHS exposure and health outcomes used self-report survey data for information on SHS. Although we adjusted for important sociodemographic factors such as occupation type, we were unable to examine the probability of SHS exposure by specific types of workplaces associated with each occupation. These limitations of the current study, together with the inevitable cross-sectional design of the KNHANES suggest the need for large and longitudinal observational studies with rigorous measurement on SHS exposure to evaluate the association of SHS exposure with baseline cardiometabolic health and follow-up data on changes in cardiometabolic health among never-smoking adult cancer survivors.

The findings of our study has public health implications concerning cardiometabolic health of never-smoking adult cancer survivors. According to the predicted trajectory on the population of cancer survivors based on the U.S Census Bureau, the number of adult cancer survivors are expected to increase dramatically and most of them are expected to be older adults over the age of 60. Thus, it is likely that increased prevalence of older adult cancer survivors will also lead to an increase in cardiovascular disease burden due to a wide range of risk factors including SHS exposure. The evidence on SHS exposure and cardiometabolic health in never-smoking adult cancer survivors found in this study could strengthen the need for smoking cessation policy-making to reduce the harmful health effect from passive smoking for the community-dwelling adult cancer survivors who are life-time never-smokers. Along with the potential mechanisms of SHS exposure on cardiometabolic health, this could promote public health awareness of household passive smoking as well as SHS exposure at workplace and public space.

## Conclusion

**Fig. 3:**
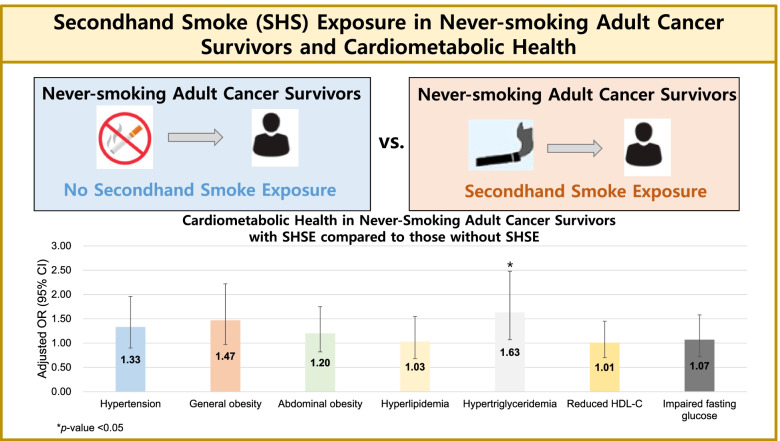
Central illustration: Association of secondhand smoke exposure and cardiometabolic health in never-smoking adult cancer survivors

Overall, our findings suggest that SHS exposure was significantly associated with higher odds of hypertriglyceridemia while nonsignificant associations were found for hypertension, obesity, worsened lipid profile, and glucose abnormalities in never-smoking adult cancer survivors as compared to those without SHS exposure (Fig. 3). This study provides evidence not only for the need for further investigation using well-designed observational studies, but also the public health imperative to review policies for minimizing SHS exposure for adult cancer survivors as well as other clinically important and vulnerable populations.

## Supplementary Information

**Additional file 1:** **Supplemental Table 1. **Multicollinearity test for independent variables measured by the variance inflation factor for the variables used to investigate the association of secondhand smoke exposure with cardiometabolic health among never-smoking adult cancer survivors in the Korea National Health and Nutrition Examination Survey, 2013-2018.

**Additional file 2:** **Supplemental Table 2. **Interaction *p*-values for each outcome in the stratified analysis for cardiometabolic health among never-smoking adult cancer survivors with secondhand smoke exposure compared to those without in the Korea National Health and Nutrition Examination Survey, 2013-2018.

## Data Availability

No additional data available. The Korea Disease Control and Prevention Agency (KDCA) provides the Korea National Health and Nutrition Examination Survey (KNHANES) to only authorized researchers upon request. Information regarding data availability can be found on the KCDA website (https://knhanes.kdca.go.kr/knhanes).
