# Supplementary material for: Association of secondhand smoke exposure with cardiometabolic health in never-smoking adult cancer survivors: a population-based cross-sectional study
Source: BMC Public Health. 2022 Mar 17;22:518. doi: 10.1186/s12889-022-12962-y (PMC8928622; doi:10.1186/s12889-022-12962-y)
Supplement: Supplementary file 1 — Additional file 1: Supplemental Table 1. Multicollinearity test for independent variables measured by the variance inflation factor for the variables used to investigate the association of secondhand smoke exposure with cardiometabolic health among never-smoking adult cancer survivors in the Korea National Health and Nutrition Examination Survey, 2013-2018. [file 12889_2022_12962_MOESM1_ESM.docx]

**Supplemental Table 1.** Multicollinearity test for independent variables measured by the variance inflation factor for the variables used to investigate the association of secondhand smoke exposure with cardiometabolic health among never-smoking adult cancer survivors in the Korea National Health and Nutrition Examination Survey, 2013-2018

| **Variable** | **Variance Influence Factor** |
| --- | --- |
| Age | 1.915 |
| Sex | 1.130 |
| Education level | 1.798 |
| Household income | 1.508 |
| Marital status | 1.053 |
| Insurance type | 1.017 |
| Occupation type | 1.206 |
| Residential area | 1.103 |
| Aerobic exercise | 1.101 |
| Muscle strengthening exercise | 1.074 |
| Total energy intake | 1.028 |
| Alcohol consumption | 1.114 |
| Family history of cardiovascular disease | 1.02 |
