# Supplementary material for: Association of secondhand smoke exposure with cardiometabolic health in never-smoking adult cancer survivors: a population-based cross-sectional study
Source: BMC Public Health. 2022 Mar 17;22:518. doi: 10.1186/s12889-022-12962-y (PMC8928622; doi:10.1186/s12889-022-12962-y)
Supplement: Supplementary file 2 — Additional file 2: Supplemental Table 2. Interaction p-values for each outcome in the stratified analysis for cardiometabolic health among never-smoking adult cancer survivors with secondhand smoke exposure compared to those without in the Korea National Health and Nutrition Examination Survey, 2013-2018. [file 12889_2022_12962_MOESM2_ESM.docx]

**Supplemental Table 2.** Interaction *p*-values for each outcome in the stratified analysis for cardiometabolic health among never-smoking adult cancer survivors with secondhand smoke exposure compared to those without in the Korea National Health and Nutrition Examination Survey, 2013-2018

|  | **Hypertension (2017 AHA/ACC^a^)** | **General obesity^b^** | **Abdominal obesity^b^** | **Hyperlipidemia^c^** | **Hypertriglyceridemia^c^** | **Reduced**  **HDL-C^c^** | **Impaired fasting glucose (ADA/WHO^d^)** |
| --- | --- | --- | --- | --- | --- | --- | --- |
| Age | <0.001 | <0.001 | 0.143 | <0.001 | <0.001 | <0.001 | <0.001 |
| <65 y |  |  |  |  |  |  |  |
| ≥65 y |  |  |  |  |  |  |  |
| Sex | 0.539 | 0.508 | <0.001 | 0.534 | 0.573 | 0.559 | 0.569 |
| Male |  |  |  |  |  |  |  |
| Female |  |  |  |  |  |  |  |
| Education level | <0.001 | <0.001 | 0.143 | <0.001 | <0.001 | <0.001 | <0.001 |
| University/College |  |  |  |  |  |  |  |
| ≤High school |  |  |  |  |  |  |  |
| Household income | <0.001 | 0.002 | 0.002 | 0.003 | 0.003 | 0.003 | 0.003 |
| Upper half |  |  |  |  |  |  |  |
| Lower half |  |  |  |  |  |  |  |
| Occupation type | 0.149 | 0.143 | 0.146 | 0.15 | 0.153 | 0.15 | 0.15 |
| Manager, professionals |  |  |  |  |  |  |  |
| Manual labor |  |  |  |  |  |  |  |
| Unemployed |  |  |  |  |  |  |  |

NOTES: adjusted odds ratios and 95% confidence intervals for each outcome is noted in Table 3.

Acronyms: AHA, American Heart Association; ACC, American College of Cardiology; BMI, body mass index; WC, waist circumferce; HDL-C, high-density lipoprotein cholesterol; ADA, American Diabetes Association; WHO, World Health Organization; SBP, systolic blood pressure; DBP, diastolic blood pressure; FSG, fasting serum glucose

^a^Defined as SBP≥130 mmHg or DBP≥80 mmHg or taking antihypertensive drugs according to the 2017 AHA/ACC high blood pressure guidelines

^b^General obesity defined as BMI ≥25.0 kg/m^2^ and abdominal obesity defined as WC≥90 cm for men and WC≥85cm for women

^c^Hyperlipidemia defined as total cholesterol ≥240 mg/dL or taking cholesterol lowering drugs; hypertriglyceridemia defined as triglyceride ≥200 mg/dL or taking cholesterol lowering drugs: reduced HDL-C defined as HDL-C≤40 mg/dL for men and HDL-C ≤50 mg/dL for women

^d^Defined as FSG of 110–125 mg/dL according to the ADA and WHO
